# Potential effects of climate change on members of the Palaeotropical pitcher plant family Nepenthaceae

**DOI:** 10.1371/journal.pone.0183132

**Published:** 2017-08-17

**Authors:** Laura K. Gray, Charles Clarke, G. R. William Wint, Jonathan A. Moran

**Affiliations:** 1 Department of Renewable Resources, University of Alberta, Edmonton, Alberta, Canada; 2 Australian Tropical Herbarium, James Cook University Cairns Campus, Cairns, Queensland, Australia; 3 Spatial Ecology and Epidemiology Group, Department of Zoology, University of Oxford, Oxford, United Kingdom; 4 Environmental Research Group Oxford (ERGO), Department of Zoology, University of Oxford, Oxford, United Kingdom; 5 School of Environment and Sustainability, Royal Roads University, Victoria, British Columbia, Canada; University of New England, AUSTRALIA

## Abstract

Anthropogenic climate change is predicted to have profound effects on species distributions over the coming decades. In this paper, we used maximum entropy modelling (Maxent) to estimate the effects of projected changes in climate on extent of climatically-suitable habitat for two *Nepenthes* pitcher plant species in Borneo. The model results predicted an increase in area of climatically-suitable habitat for the lowland species *Nepenthes rafflesiana* by 2100; in contrast, the highland species *Nepenthes tentaculata* was predicted to undergo significant loss of climatically-suitable habitat over the same period. Based on the results of the models, we recommend that research be undertaken into practical mitigation strategies, as approximately two-thirds of *Nepenthes* are restricted to montane habitats. Highland species with narrow elevational ranges will be at particularly high risk, and investigation into possible mitigation strategies should be focused on them.

## Introduction

The monotypic Palaeotropical family Nepenthaceae (genus *Nepenthes*) is the largest family of pitcher plants, comprising approximately 140 species. Nutrients from prey captured by the pitchers are used to supplement those taken up from the nutrient-poor substrates on which *Nepenthes* predominantly occur [1]. The Nepenthaceae has an extensive geographical range across the Palaeotropics, from Madagascar through Southeast Asia, to New Caledonia; the centre of diversity encompasses the islands of Borneo, Sumatra, and the Philippines. The majority of *Nepenthes* species fall into two rather broad groupings, defined largely by their altitudinal distributions: so-called "lowland" species occur in habitats ranging from 0 m to approximately 1100 m above sea level (asl), whereas "highland" species are restricted to cooler, montane habitats, usually above 1100 m asl [2–3]. Beyond these two major groups, a few species occupy intermediate altitudinal ranges from approximately 700 to 1400 m asl; in addition, some species can occasionally be found growing above or below their typical altitudinal thresholds. In general terms, the Nepenthaceae can be considered a largely highland family, as approximately two-thirds of the currently-recognized species occur at altitudes > 1100 m asl [3].

As a genus, *Nepenthes* is characterized by narrow endemism: the majority of species have very small extents of occurrence. More than half of the highland species are either confined to a single mountain summit, or occur on a small number of peaks within a single mountain range. By contrast, many of the lowland species have extensive ranges. For example, *Nepenthes mirabilis* (Lour.) Rafarin extends throughout the lowlands of Southeast Asia, as far eastwards as New Guinea and northern Australia. Another widespread lowland species is *Nepenthes rafflesiana* Jack, which is abundant on nutrient-deficient substrates in the lowlands of the Malay Peninsula, Borneo, and parts of Sumatra. However, some lowland species also have highly circumscribed ranges. For example, *Nepenthes campanulata* Sh. Kurata is an obligate limestone endemic from Borneo, and occurs in a small geographical area determined by the very limited distribution of a particular type of limestone formation [4]. Although there appear to be multiple drivers of narrow endemism within *Nepenthes* (few of which are well-understood), the overwhelming pattern is one of species being confined to relatively small geographical areas, where they occupy specialized niches. Unfortunately, narrow endemics are thought to be at greater risk from stochastic and deterministic disturbances than widespread species [5]. Many parts of Southeast Asia, the insular regions in particular, continue to experience unprecedented levels of anthropogenic habitat disturbance and destruction [6]. Thus, there is an urgent need to assess the consequences of these threats to narrow endemic species.

Anthropogenically-driven climate change is one such threat, and is expected to have profound effects on ecosystems worldwide in the coming century. Climate-driven changes in species distributions, community structure and vegetation dynamics, are already being observed in several ecosystems [7–9]. Tropical montane ecosystems (including the cloud forests that support more than half of all known *Nepenthes* species) house a substantial proportion of global biodiversity, and are at particularly high risk of species extinction [10–14].

Given the established vulnerability of tropical highland taxa to the effects of climate change, the current study was undertaken to compare the possible consequences of these effects on the extent of climatically-suitable habitat for two *Nepenthes*—one highland and one lowland species—from the island of Borneo. Specifically, we posed the following question: How will predicted anthropogenically-driven climate change affect the areas of climatically-suitable habitat for the two species examined?

## Materials and methods

### Study species

The two Bornean species included in this study where chosen to represent lowland and montane *Nepenthes*. The widespread lowland species *N*. *rafflesiana* has an altitudinal range between 0 and *ca*. 1200 m asl, although the vast majority of populations occur below 100 m asl [2]. *Nepenthes tentaculata* Hook. f. is a highland species, inhabiting mossy forest between 700 and 2400 m asl, with the vast majority of localities lying above 1200 m asl [2]. In Borneo, it is widely distributed in suitable montane habitat.

Geographical coordinates (10 m accuracy) were obtained by direct observation of populations of both species in the field by one of the authors (CC) from 1987–2012, supplemented with herbarium records. For the latter, locality data were obtained from the Global Biodiversity Information Facility (http://www.gbif.org/), with erroneous records and localities outside Borneo being discarded. For *N*. *rafflesiana*, 36 records were retrieved, and 25 retained. Added to the original field observations (CC), this gave a total of 164 localities; for *N*. *tentaculata*, 55 points were retrieved and 38 retained. Combined with the original field observations, this gave a total of 54 localities. The retained records were cross-checked against personal observations of locations of both species made in the field by CC from 1987 to 2012. Only records that have been ground truthed were used in the study. The Maxent modelling software (see below) treated all data points within a 1 km polygon as a single datum. Therefore, the total effective numbers of localities were 139 and 52 for *N*. *rafflesiana* and *N*. *tentaculata*, respectively. The location data are available in S1 and S2 Datasets.

### Climate data

Nineteen bioclimatic variables were used as predictors of suitable habitat [15]; we chose to use the Bioclim variables rather than meteorological data (e.g. mean monthly rainfall or temperature), as the former have been shown to be more useful for describing the meteorological constraints on the distributions of species [16–18], and have been used successfully to model distributions of many plant taxa [19–22]. They are publicly available as global climate grids at 30 arc-seconds (~ 1 km) resolution from the WorldClim dataset Version 1.4 (http://www.worldclim.org/bioclim) [15, 23]. The variables are summarized in Table 1.

**Table 1:** Maxent model results.

Bioclimatic Variable		Permutation Importance (%) ^a^	
		*N*. *rafflesiana* (*n* = 139)	*N*. *tentaculata* (*n* = 52)
Bio1	Annual mean temperature	0	0
Bio2	Mean diurnal temperature range	1.72	0.39
Bio3	Isothermality ^b^	0.15	0
Bio4	Temperature seasonality ^c^	8.36	0.64
Bio5	Max. temperature of warmest month	0	0
Bio6	Min. temperature of coldest month	0	30.57
Bio7	Annual temperature range ^d^	2.99	0
Bio8	Mean temperature of wettest quarter	1.23	0
Bio9	Mean temperature of driest quarter	2.30	0.04
Bio10	Mean temperature of warmest quarter	26.50	0
Bio11	Mean temperature of coldest quarter	0	37.85
Bio12	Annual precipitation	0.02	22.31
Bio13	Precipitation of wettest month	0.34	5.90
Bio14	Precipitation of driest month	6.66	0.25
Bio15	Precipitation seasonality ^e^	3.44	0.07
Bio16	Precipitation of wettest quarter	30.23	0
Bio17	Precipitation of driest quarter	8.00	0.76
Bio18	Precipitation of warmest quarter	4.80	0.64
Bio19	Precipitation of coldest quarter	3.26	0.58
Test AUC ^f^		0.88	0.97

^a^ % decrease in training AUC resulting from randomly permuting values of a given variable during the training phase of model development.

^b^ (Bio2/Bio7)*100.

^c^ (Standard deviation)*100.

^d^ (Bio5-Bio6).

^e^ Coefficient of variation of precipitation.

^f^ Area under curve. A value of 1 represents a perfect fit of the model.

### Maxent modelling and evaluation

We used Maxent v. 3.3.3k [24–26] to model habitat suitability on the island of Borneo independently for each species. Model outputs present an estimate of the probability of presence for a given species between 0 and 1 for each 30 arc-second pixel, with lower values indicating that the pixel represents less suitable habitat. Sample size for *N*. *tentaculata* was below the minimum of 80 observations necessary for the use of product or threshold feature types [27]; therefore, we adopted a conservative approach, using only linear and quadratic features for our model execution for both species. By excluding product features, we created a more easily-interpreted additive model [27].

In order to allow the model a sufficient number of iterations for convergence, we increased the maximum number of iterations from 500 (the default) to 5000. This reduced the likelihood of Maxent either over- or under-predicting habitat suitability. In addition, we accounted for potential geographic sampling bias in the data (which may have occurred due to differing degrees of accessibility between sampling locations), by selecting pseudo-absences from background data with a similar sampling bias to the occurrence data [28]. Bias layers for each species were constructed using the smallest administrative unit for each of the three countries on the island of Borneo (Malaysia, Brunei and Indonesia); an administrative unit was selected for the bias file if a species observation occurred within its boundary [29]. The inclusion of the bias file in each species model ensured that the default 10,000 pseudo-absences used in model calculations were selected from the small-scale geographic units in which species observations were recorded, and not from the whole study area.

The performance of each species model was evaluated by setting the random test percentage to 30; seventy percent of the observations were randomly selected as a training data set and the remaining 30% were used to test the model. In order to estimate the robustness of model outputs, the replicates option was set at 10, so that the selection of training and test data from the species observations, as well as the calculation of performance statistics, were repeated. Model fit was evaluated as the average area under the curve (AUC) of the receiver operating characteristic (ROC) across all ten model replicates. AUC is a widely-used, threshold-independent measure of model evaluation, which represents the probability that the model classifier will correctly identify a randomly-chosen true presence [30–31]. AUC values range from 0 to 1, with a value of 0.5 representing a model that correctly identifies true species occurrences equal to a random guess, and a value of 1 indicating a perfect model fit. While an alternative jackknife or "leave-one-out" procedure has been suggested to evaluate model accuracy for small sample sizes [32], the number of observations for both species in the current study is at least one and a half times greater than in the study for which that procedure was recommended. Thus, to prevent overoptimistic estimates of predictive power, we opted to use the standard AUC calculations.

For examination of which bioclimatic variables were driving the models, we used "Permutation Importance". This represents a measure of decrease in training AUC resulting from randomly permuting values of that variable during the training phase of model development; thus, the decrease in training AUC is proportional to the importance of that variable to the model [25]. All modified parameters listed above were common across each species' Maxent model, with the exception of the bias files.

### Model future projections

Using Maxent, the models for each species were projected onto climate surfaces for the 2020s, 2050s, and 2080s, which represent forecasted climate trends over individual 30-year periods (2001–2040, 2041–2070, and 2071–2100). These surfaces were obtained from the EDENext Data Management Team (EDENext DMT; http://www.edenextdata.com/). For each time period, the EDENext DMT utilized climate projections generated from WorldClim climate grids at 30 arc-seconds (~ 1km) resolution, prepared by four modeling groups for the IPCC 4th Assessment [33], and incorporating three SRES emission scenarios: A1b, A2s, and B2a [34–35]. The modeling groups included HADLEY (Hadley Centre of the UK Met Office, United Kingdom), CSIRO (Commonwealth Scientific and Industrial Research Organization, Australia), CCCMA (Canadian Centre for Climate Modelling and Analysis), and CCSR/NEIS (Center for Climate System Research and National Institute for Environmental Studies, Japan). To account for the variation in projected climate and provide a consensus average set of projections, EDENext DMT developed composite ensemble grids across all modelling groups and emission scenarios for each bioclimatic variable in each time period, using the mean value of the inputs for each pixel. Graphical outputs were produced using ArcMap v. 10.1 (Esri Inc., Redlands, CA, USA).

## Results

### Model evaluation

Model evaluation statistics for both species are presented in Table 1. Mean AUC values from model replicates were high for both species (0.88 and 0.99). The AUC value is a measure of model accuracy, where, for instance, a value of 0.88 indicates that 88% of the time a random sample from presence predictions will have a greater probability of presence than a random selection from absence predictions across all available probability thresholds. In general, AUC values greater than 0.7, 0.8, and 0.9 are considered fair, good and excellent, respectively [31].

Results from the Permutation Importance analyses (Table 1) show that the model for the lowland *N*. *rafflesiana* is driven primarily by mean temperature of the warmest quarter (26.5%) and precipitation of the wettest quarter (30.23%). In contrast, the model for the highland *N*. *tentaculata* is driven primarily by minimum temperature of the coldest month (30.57%), mean temperature of the coldest quarter (37.85%), and annual precipitation (22.31%).

*N*. *tentaculata* shows a higher model accuracy than *N*. *rafflesiana* (Table 1). These results are likely to be related to the more limited range of the former, which is a highland species: many studies have found that species distribution models for species with smaller ranges or narrower ecological tolerances have higher prediction accuracy than models developed for more widespread species [36–39]. These studies conclude that the higher predictive accuracy is a result of the fact that it is easier to separate suitable from unsuitable habitat for species with restricted environmental tolerances or very specific habitat requirements. In addition, although anecdotal accounts of additional Bornean *N*. *rafflesiana* and *N*. *tentaculata* populations exist, we did not have sufficiently accurate geographical coordinate data to include them in the current study. Consequently, the estimated ranges of both these species are somewhat conservative and exclude patches of currently suitable, occupied habitat elsewhere in Borneo. This confers a high degree of conservatism upon the current and predicted distributions of suitable habitat.

**Fig 1:**
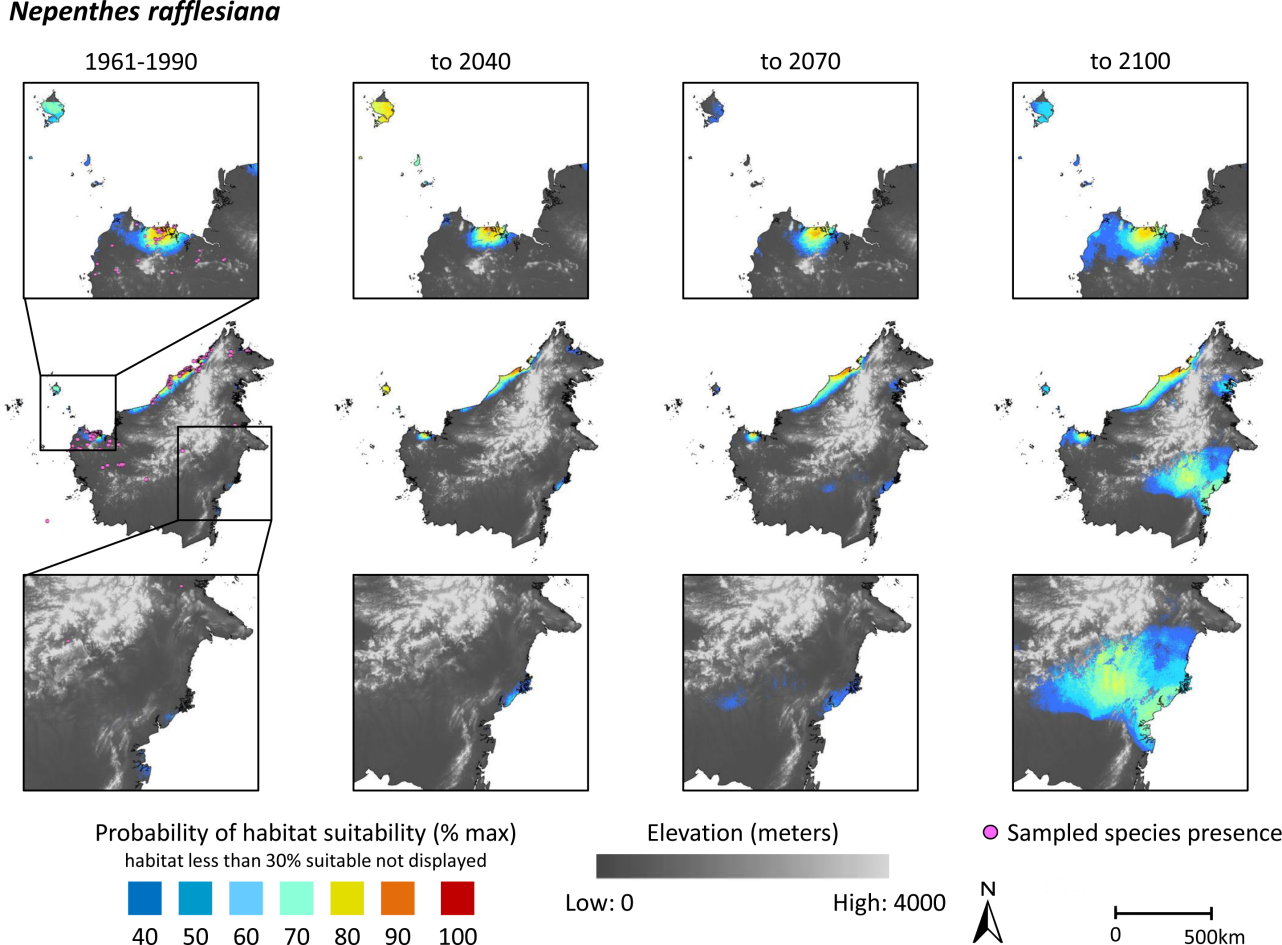
Modeled habitat suitability on the island of Borneo for the lowland species *Nepenthes rafflesiana*, using Maxent (*n* = 139 localities). Habitat suitability is presented for the periods 1960–1990 (left panel), 2011–2040 (second panel), 2041–2070 (third panel), and 2071–2100 (right panel). Model results are overlaid onto an elevation map for illustration purposes. Probability of habitat suitability, ranging from 0 to 1, is plotted on a color scale, with orange representing values of 0.3–0.4, and dark blue representing values of 0.9–1. Areas with < 30% probability of habitat suitability are not illustrated. Inset boxes show higher resolution maps for areas of high occurrence. Scale bar refers to center row of images.

**Fig 2:**
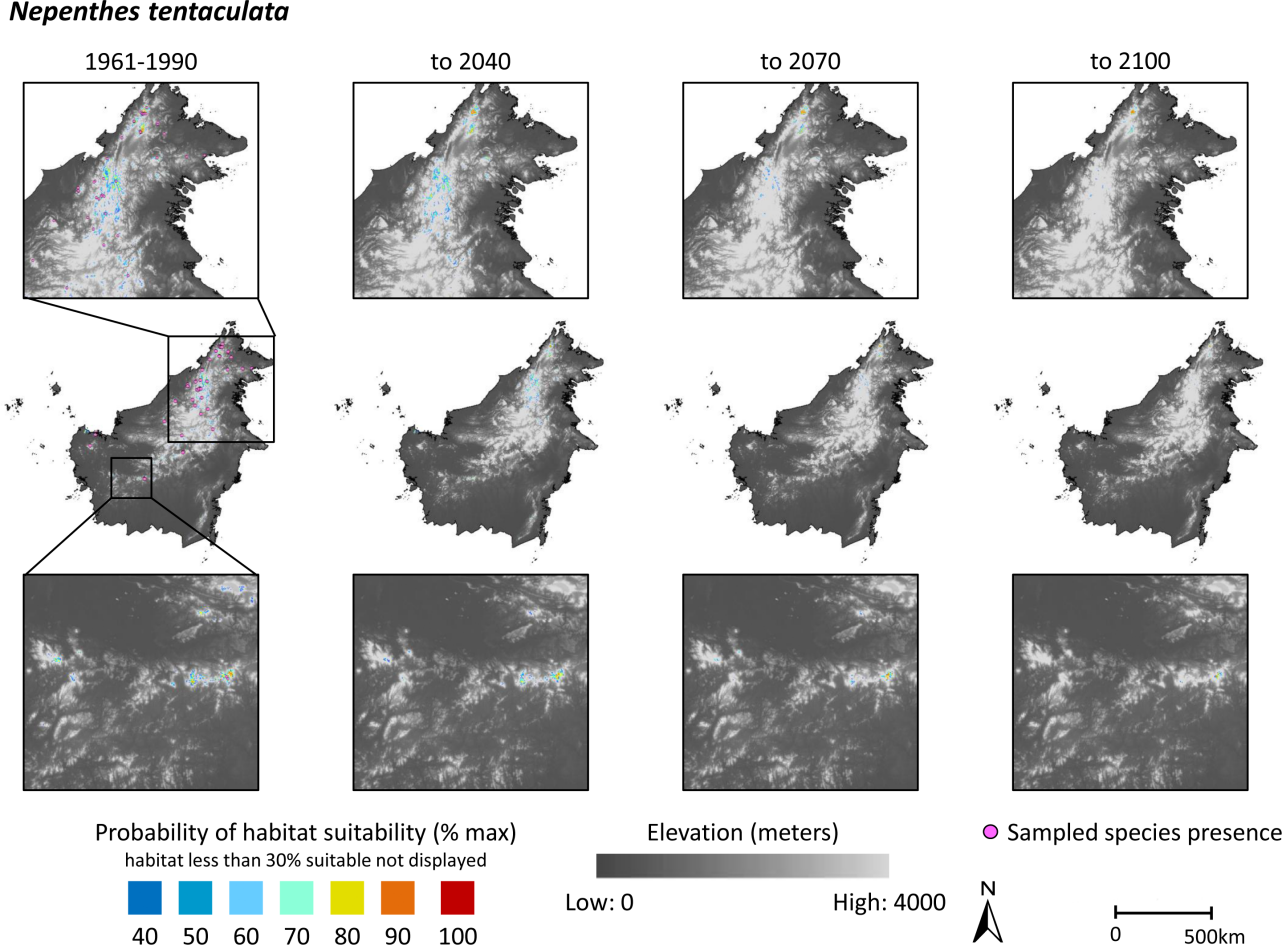
Modeled habitat suitability on the island of Borneo for the montane species *Nepenthes tentaculata*, using Maxent (*n* = 52 localities). Habitat suitability is presented for the periods 1960–1990 (left panel), 2011–2040 (second panel), 2041–2070 (third panel), and 2071–2100 (right panel). Model results are overlaid onto an elevation map for illustration purposes. Probability of habitat suitability, ranging from 0 to 1, is plotted on a color scale, with orange representing values of 0.3–0.4, and dark blue representing values of 0.9–1. Areas with < 30% probability of habitat suitability are not illustrated. Inset boxes show higher resolution maps for areas of high occurrence. Scale bar refers to center row of images.

While the sampling points do appear clustered in some areas (Figs 1 and 2), spatial autocorrelation was not deemed a significant concern in the current study. It has been argued that spatial autocorrelation in species distribution models is a concern when, for example, ecological or evolutionary processes prevent taxa from shifting their distributions [40]. For both *N*. *rafflesiana* and *N*. *tentaculata*, their relatively widespread observed occurrences suggest that such processes have not exerted strong constraints on these species shifting their distributions over time.

### Predicted habitat suitability and changes in area of climatically suitable habitat

**Fig 3:**
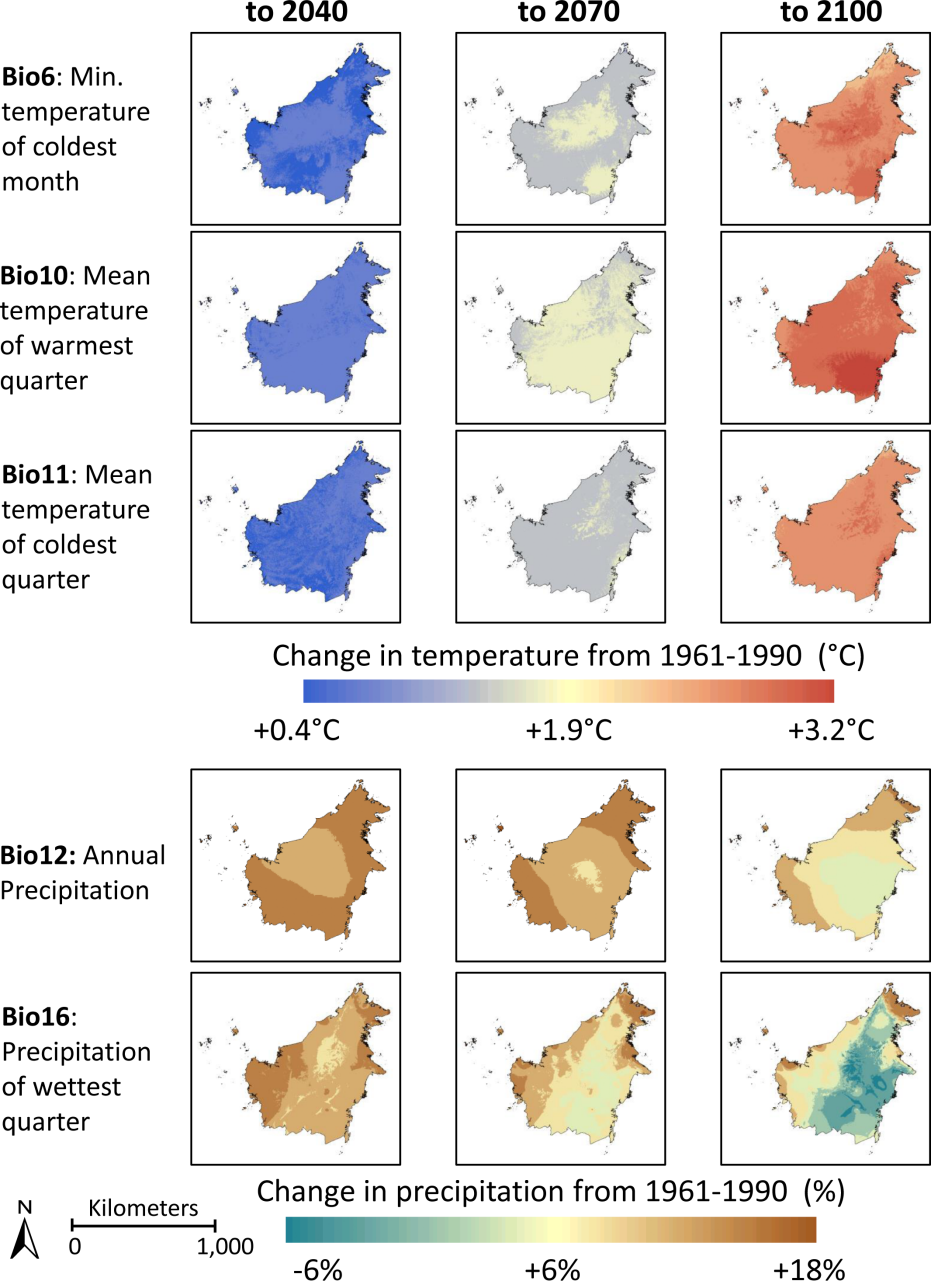
Projected changes in important Bioclim variables on the island of Borneo identified in Table 1. Images represent changes in Bioclim variables relative to the 1961–1990 climate surfaces, projected to 2040, 2070, and 2100. Changes in temperature variables (Bio6, Bio10, and Bio11) are presented as °C; changes for precipitation variables (Bio12, Bio16) are presented as percentages.

Graphical representations of modeled habitat suitability are presented in Figs 1 and 2 for *N*. *rafflesiana* and N. *tentaculata*, respectively. Modeled habitat suitability is presented for the periods 1960–1990 (current climate surfaces), 2011–2040, 2041–2070, and 2071–2100 (EDENext projections). The model results have been overlaid onto an elevation layer from the SRTM 90 m resolution Digital Elevation Database v. 4.1 [41]. The elevation layer was not used as a predictor variable in the Maxent modelling, and has been included in the Figs solely to illustrate the topographic characteristics of locations where each of the species currently occurs or may occur in the future. Probability of habitat suitability, ranging from 0 to 1, is plotted on a color scale, where orange represents values of 0.3–0.4 and dark blue represents values of 0.9–1. Areas with < 30% probability of being suitable habitat are not illustrated. The inset boxes show higher resolution maps for areas of high occurrence for each species. The estimated effects of climate change on variables identified by Permutation Importance analysis as driving the models are presented in Fig 3.

Projected changes in area of climatically-suitable habitat (defined here as area denoting habitat suitability of ≥60%) are presented in Table 2. The 60% value represents a conservative approach [42]. *N*. *rafflesiana* is predicted to experience a small decrease in climatically-suitable area to 2040 and 2070 (-8 and -11%, respectively), then an increase of 111% by 2100. Climatically-suitable habitat for *N*. *rafflesiana* is constrained primarily by precipitation of the wettest quarter and mean temperature of the warmest quarter (Table 1). Comparisons of shifts in these two bioclimatic variables over our projection periods show that habitat losses along the northwestern coast are likely associated with an increase in mean temperature of warmest quarter of approximately 2°C in that region (Fig 3). Conversely, the projected increase in precipitation of the wettest quarter by 2100 in the southeastern corner of Borneo corresponds to where the expansion of climatically-suitable habitat for *N*. *rafflesiana* is the greatest (Fig 3). In contrast to *N*. *rafflesiana*, climatically-suitable habitat for *N*. *tentaculata* is predicted to undergo a 50% reduction by 2040, and an 89% reduction by 2100 (Table 2).

**Table 2:** Predicted net % change in area of climatically-suitable habitat relative to the 1961–1990 climate surfaces, for the periods 2011–2040, 2041–2070, and 2071–2100 for *N*. *rafflesiana* and *N*. *tentaculata*.

Species	Net change in area of climatically-suitable habitat (%) ^a^		
	to 2040	to 2070	to 2100
*N*. *rafflesiana*	-8	-11	+111
*N*. *tentaculata*	-50	-82	-89

^a^ Climatically-suitable habitat is defined as area denoting habitat suitability of ≥60%.

## Discussion

### Comparison of predicted effects on lowland and highland *Nepenthes* species

A recent review of the threats to carnivorous plants worldwide cited habitat loss to agriculture as the dominant anthropogenic pressure; other threats included over-collection of specimens, and pollution [43]. Neither species in the current study are significantly threatened by over-collection, land clearing or logging: the soils that support *N*. *rafflesiana* are too nutrient-poor to support agriculture, and much of the range occupied by *N*. *tentaculata* is in topography that precludes extensive logging, or in forest formations that are not desirable for harvest (CC, pers. obs.). However, our results suggest that climate change should be added to the list of threats to montane *Nepenthes* species. The degree of risk posed by climate change will be dependent to a large degree upon the elevational distributions of individual species and the characteristics of the niches they occupy on high mountain summits and ridges.

In the case of the lowland *N*. *rafflesiana*, the predicted increase in climatically-suitable habitat by 2100 based on climate projections needs to be viewed with a degree of caution. This species is not encountered in the lowland dipterocarp forests that typify the original natural cover of much of Southeast Asia; the requirements for botanical carnivory (low soil nutrient availability, high water availability and high incident radiation [44]) are seldom met in these forests [3]. Thus, much of the climatically-suitable habitat predicted by the model will be unavailable for colonization, due to edaphic and other constraints (however, see below for an exception to this general rule). *N*. *rafflesiana* is restricted largely to heath forest formations that have developed on acidic, nitrogen-poor sandy soils [2,45], and many—though by no means all—of these formations occur in low-lying, coastal areas. Projected increases in sea level as a result of climate change are predicted to have significant negative effects on the extent of coastal land in Borneo and other parts of Southeast Asia [46–47]. In a recent study, it was estimated that coastal land losses of 1% and 15% might be expected for Borneo and the Philippines, respectively, in the event of a 1m rise in sea level [48]. Indeed, islands are at particularly high risk of inundation: a recent analysis of the exposure of island biodiversity hotspots to this particular risk, identified the islands of Sundaland (Borneo, Sumatra, and Java) and the Philippines as particularly vulnerable in this respect [49]. This region circumscribes the centre of diversity for *Nepenthes*. Thus, species that are restricted to the coastal lowlands of this area may be at risk of habitat loss, despite favorable future trends in suitable climate. That said, *N*. *rafflesiana* occurs up to *ca*. 1100 m asl, as do the majority of the lowland Bornean *Nepenthes* [3], and so is unlikely to face extinction as a direct result of sea level rise. Furthermore, the creation of new nutrient-deficient substrates, due to the removal of nutrient-rich top soil when lowland forest formations are cleared for development, can give rise to a vegetation type known as "Adinandra belukar", which resembles heath forest in many respects and supports many species that naturally occur in that forest type [50]. In many parts of Malaysia, Adinandra belukar is aggressively colonized by *N*. *rafflesiana*, as well as by *Nepenthes gracilis* Korth. Recent field observations conducted by CC demonstrate that *N*. *rafflesiana* is now conspicuous at a number of locations where it was rare or absent in the 1980s.

With regards to the montane *N*. *tentaculata*, the predictive models generated in the current study should come as little surprise, as similar reductions in suitable habitat for montane species have been predicted across multiple taxa and geographic regions [51–54], and several studies have demonstrated changes in altitudinal distributions of plant and animal species at multi-decadal or even decadal scales [8,12,55–56].

### The effects of elevational range

**Fig 4:**
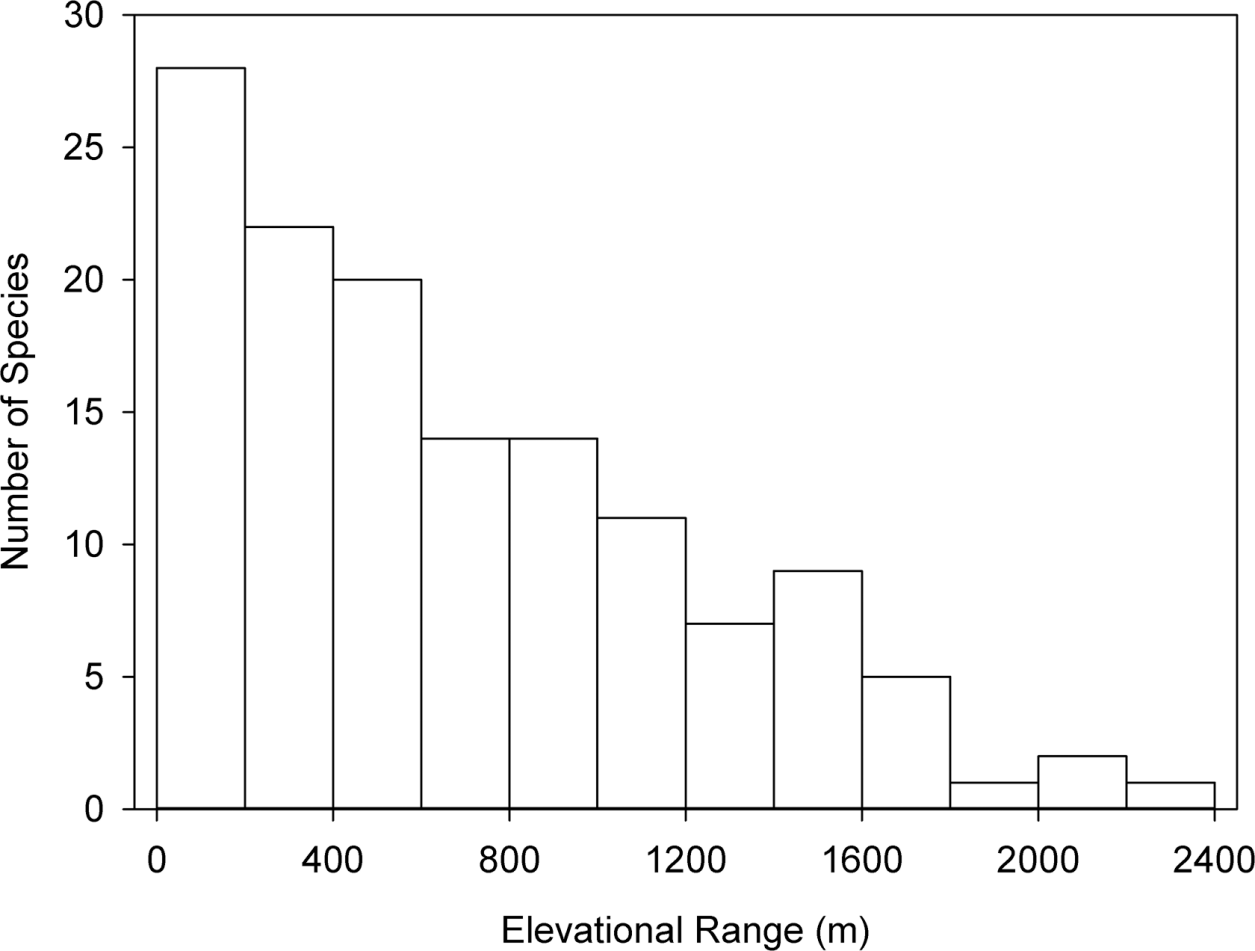
Frequency histogram of elevational range (ER) for 134 *Nepenthes* species. Data from Clarke and Moran (2015) [3].

Elevational range (ER) is the breadth of altitude encompassing the distribution of a species (i.e., the range between the lowest and highest elevations at which the species occurs), and has an effect on the vulnerability of montane taxa to climate change. For example, one recent study identified a strong negative relationship between ER and extinction risk in birds [57]. Elevational range can be thought of as providing an inverse measure of thermal specialization, and likely reflects the degree of inherent plasticity of a species (physiological and/or behavioral) in response to fluctuations in ambient conditions [58]. An exception to this may be species that are restricted to the summit of one mountain, as it is not possible to test whether or not the low ER for such species is due to lack of plasticity with regards to climatic conditions, or lack of upslope habitat. A frequency histogram of ERs of *Nepenthes* species is presented in Fig 4. It can be seen that the distribution is skewed heavily towards narrow ranges: out of 134 species, 28 (21% of the total) are confined to ranges ≤ 100 m, and 70 species (52%) have ranges of ≤ 500 m. It must be stressed that not all of the *Nepenthes* species with narrow ERs are montane; however, those that are, are likely to be subject to greater risk of extinction due to climate change. In this respect, several montane species appear to be at particularly high risk, including *Nepenthes macrophylla* (Marabini) Jebb & Cheek, which occurs only around the summit of Mount Trusmadi (ER *ca*. 450 m; JM pers. obs.), *Nepenthes deaniana* Macfarl, a species that is endemic to Thumb Peak (ER 110 m; CC pers. obs.), and *Nepenthes murudensis* Culham ex Jebb & Cheek, which is endemic to Mount Murud (ER 300 m [2]). Although high mountains such as Mount Kinabalu (Borneo, 4095 m asl) and Mount Doorman (New Guinea, 3557 m asl), may serve as refugia for montane endemic *Nepenthes* species during periods of elevated global temperatures, lower mountains are liable to act as "summit traps", which prevent sufficient uphill migration towards climatically-suitable zones to allow populations to persist [59]. *Nepenthes* species that are endemic to low mountains are thus at particular risk in a warming climate. The most prominent examples of this are in the mountains of Palawan (e.g., Mt. Victoria (*Nepenthes attenboroughii* A.S. Rob., S. McPherson & V.B. Heinrich), Thumb Peak (*N*. *deaniana*) and Sultan Peak (*Nepenthes palawanensis* S. McPherson, Cervancia, Chi. C. Lee, Jaunzems, Mey & A.S. Rob.)), the majority of which are < 1500 m in height.

### Limitations of the models

It is important to acknowledge the potential limitations on model accuracy imposed by an analysis carried out at 30 arc-second resolution in areas of high topographic variability. A 30 arc-second polygon (~ 1 km^2^) projected onto mountainous terrain, will almost certainly encompass a heterogeneous mix of highly-localized microhabitats that have been shaped by unique combinations of aspect, slope, prevailing winds, etc. Not all of these microhabitats will necessarily be suitable for a given highland *Nepenthes* species, many of which are almost exclusively confined to stunted vegetation on mountain summits and ridge tops (CC, pers. obs.). Thus, it is feasible that the area of habitat occupancy of a montane narrow endemic *Nepenthes* may be constrained not only by climatic conditions, but by highly localized environmental factors and processes that operate at levels below the scale of a 30 arc-second polygon. This has two broad implications: first, suitable niche habitats that are extremely restricted in area may not be detected and/or could persist (or arise) at spatial scales that are too fine to be captured by the model; and second, other environmental factors and processes may operate at very fine spatial scales and may have more or less influence over the distribution of a species than the temperature and rainfall patterns that can be detected at 30 arc-second resolution. Despite these potential sources of error, we believe that the models nonetheless provide valid predictions of the *overall* patterns regarding changes in the extent of climatically-suitable habitat.

## Conclusions

The results of the current study suggest a rather bleak outlook for *N*. *tentaculata*, and a similar future could await several other montane *Nepenthes* species from the higher mountains of Borneo, such as *Nepenthes lowii* Hook.f., *Nepenthes rajah* Hook.f., *Nepenthes villosa* Hook. f. and *N*. *macrophylla*. Although the current study uses Bornean examples, it is important to note that the findings are applicable to *Nepenthes* species endemic to mountainous areas throughout the extensive geographical area occupied by the Nepenthaceae. It is worth reiterating the fact that the Nepenthaceae is largely a montane family, and particularly rich in cloud forest endemic species; in fact, only about one-third of *Nepenthes* species are restricted to lowland habitats [3]. Therefore, many montane endemic *Nepenthes* species outside of Borneo are potentially at risk from anthropogenic climate change. For instance, a number of species from Palawan are confined to the uppermost parts of the island's highest mountains. An example is *N*. *attenboroughii*, which is confined to the uppermost 100 m around the summit of a single mountain. Even a slight change to local temperature and rainfall patterns could be sufficient to threaten this species with extinction. By contrast, we believe that the genus as a whole is unlikely to succumb completely to the predicted effects of anthropogenically-driven climate change. *Nepenthes* are thought to have been in existence for tens of millions of years [2,60]. If this timescale is correct, the genus has likely endured equivalent or perhaps even greater climatic upheavals than those currently predicted.

In the long term, uphill forcing of montane species due to climate change will help to drive allopatric speciation. It can be expected that widely-dispersed populations on tropical mountain ranges will be forced upslope, causing them to split into discrete, thermally-isolated sub-populations [53]. This may be followed by genetic drift if the isolated populations persist for a sufficient number of generations, leading in some instances to reproductive barriers and consequent speciation [61–62]. That the same process has occurred in *Nepenthes* in the past, is suggested by the current distribution of closely-related Bornean montane species such as *N*. *villosa* and *N*. *macrophylla*. The former occurs on Mount Kinabalu and Mount Tambuyukon, while the latter is restricted to Mount Trusmadi, 55 km to the south. A similar example is provided by the closely-related Bornean species *N*. *lowii* and *Nepenthes ephippiata* Danser [3]. Such separation into small subpopulations via uphill migration during past periods of elevated global temperatures, is probably responsible for the high degree of montane endemism currently observed in *Nepenthes*. However, this long-term prospect will be scant consolation for observers in the coming century, as several montane *Nepenthes* species with restricted ERs face increased risk of extinction, unless measures are taken to address the problem.

### Possible future actions

We believe that there are a number of possible courses of action with regards to highland *Nepenthes* species with narrow ERs. These are not mutually exclusive, and a combination of *in situ* and *ex situ* approaches might be required to maintain sufficient genetic variability to allow persistence of a given species [63]. The possible options include:

1. Collection of seeds of vulnerable species for long term storage in germplasm banks, planned so as to collect representative genetic variation of endangered populations. This *ex situ* approach has been suggested for vulnerable Mexican *Quercus* spp. and *Pinus* spp., as well as South American montane endemic plant species [51,63]. There are a number of large-scale seed banks in operation, such as the Millennium Seedbank [64]. However, the systematic collection, preparation (e.g. *in situ* drying) and long-term storage of seeds, would require considerable logistical resources; further, the administrative and legal hurdles associated with the collection of seeds from multiple regional and state jurisdictions, would be considerable. In the absence of suitable habitat for reintroduction, this strategy alone will be less than optimal. *We emphatically do not recommend that private/individual initiatives be undertaken*;
2. Assisted upslope migration of seeds or seedlings of vulnerable highland species. Assisted migration has a highly controversial history [65–71]. Although policy with regard to assisted migration is beyond the scope of this article, a number of papers provide frameworks that may be adaptable to *Nepenthes* conservation [72–74]. Given the possibility of unintended ecological consequences from assisted migration, then if this strategy is to be considered at all, we recommend that *small-scale* trials be carried out, as has been done for North American forest tree species [75–76]. In the case of montane *Nepenthes* species that have mutualistic associations with terrestrial mammals (*N*. *lowii*, *N*. *macrophylla*, and *N*. *rajah* [77–79]), decisions regarding assisted migration will need to take into account the presence/absence of the mammalian partner(s) at sites selected for seed or seedling translocation. There is also the possibility that translocated *Nepenthes* populations may not encounter the spectrum of potential prey taxa that they have evolved to attract and capture;
3. Establishment of metapopulations that include patches of suitable habitat not currently inhabited by the target species [80]. Species that are currently restricted to the summit areas of lower mountains (and thus, potentially headed for "summit traps" [59]) would be potential candidates for this strategy. This third option would entail the establishment of new populations, for instance on mountains that do not currently support the target species. New populations established in climatically-suitable habitat would need to be close enough to other populations (existing or newly-established) to allow free gene flow, and research would need to be undertaken into seed dispersal ranges for the target species to ensure this.

However, a significant danger of this approach is the very real possibility of hybridization with congeners already present at the new sites [81]. Given the facility with which *Nepenthes* species hybridize under natural conditions [2,82], the potential for introgression exists, especially if both species flower at the same time of year. Should hybrid progeny be allowed to persist at the new site, the potential exists for the generation of a reproductively-independent hybrid swarm. Intervention to remove unwanted hybrid progeny would be time-consuming, logistically difficult, and expensive. In the case of highland *Nepenthes* that have resource-exchange mutualistic associations with small terrestrial mammals (*N*. *lowii*, *N*. *rajah*, and *N*. *macrophylla*), pitcher shape, proportions, scent and color are finely tuned to the geometry and sensory modalities of their mammalian partners [77–79,83]. It is unlikely that hybrids between these and more "typical" species would produce pitchers of the required geometry to maintain this association. Thus, introgression would result in the reduction in number of these specialist pitchers, potentially causing local collapse of the mutualistic association.

Consequently, investigations into the feasibility of this third option should focus on identification of suitable patches of future habitat within protected areas, as well as constraints on seed dispersal by target species, and possible negative consequences to the receiving ecosystem (especially the potential for hybridization). Option 3 would be the riskiest of the potential actions outlined here, and investigation into its feasibility would need to be undertaken with a commensurately high degree of caution. We include this option in order to stimulate discussion, not as an endorsement.

## Supporting information

S1 Dataset Location coordinates for *Nepenthes rafflesiana* in Borneo. (CSV)

S2 Dataset Location coordinates for *Nepenthes tentaculata* in Borneo. (CSV)
